# Supplementary material for: Analysis of PPARγ Signaling Activity in Psoriasis
Source: Int J Mol Sci. 2021 Aug 10;22(16):8603. doi: 10.3390/ijms22168603 (PMC8395241; doi:10.3390/ijms22168603)
Supplement: Supplementary file 1 [file ijms-22-08603-s001.zip › Supplemental materials_Analysis of PPARg signaling activity in psoriasis/Pathway models/Models images and html files/Model of PPARG related pathways in psoriasis (short version)/15094.html]

RASGRP1


# Protein RASGRP1

|  |  |
| --- | --- |
| URN | urn:agi-llid:10125 |
| Total Entities | 0 |
| Connectivity | 525 |
| Name | RASGRP1 |
| Description | RAS guanyl releasing protein 1 (calcium and DAG-regulated) |
| Notes | This gene is a member of a family of genes characterized by the presence of a Ras superfamily guanine nucleotide exchange factor (GEF) domain. It functions as a diacylglycerol (DAG)-regulated nucleotide exchange factor specifically activating Ras through the exchange of bound GDP for GTP. It activates the Erk/MAP kinase cascade and regulates T-cells and B-cells development, homeostasis and differentiation. Alternatively spliced transcript variants encoding different isoforms have been identified. Altered expression of the different isoforms of this protein may be a cause of susceptibility to systemic lupus erythematosus (SLE). [provided by RefSeq, Jul 2008] |
| Primary Cell Localization | Cytoplasm |

---

|  |  |
| --- | --- |
| Pathway | High Fat Diet Activates cAMP Related Exocytosis in Synapse and Endocrine Cell |
|  | Dopamine Receptors Signaling |
|  | Serotonin Receptors Signaling |
|  | CRH Secretion Regulation |
|  | Prolactin Production |
|  | Mineralcorticoids Secretion Regulation |
|  | Plasmin Effects in Inflammation |
|  | Proplatelet Maturation |
|  | Platelet Activation via GPCR Signaling |
|  | Vascular Endothelial Cell Activation by Blood Coagulation Factors |
|  | Nociception Expression Targets Signaling |
|  | Vascular Motility |
|  | NPY1R -> CRH/POMC Production |
|  | NTSR1 Expression Targets |
|  | EDNRA/B -> Vascular Motility |
|  | ADRA1 -> Vasoconstriction |
|  | ADRA1A -> IL6 Production |
|  | ADRA2C/ADRB2 -> Vasoconstriction |
|  | ADRA2C/ADRB2 -> Synaptic Endocytosis |
|  | ADRA1 -> Prostaglandin Generation |
|  | BDKRB1/2 -> Prostaglandin Generation |
|  | CNR1/2 -> IL1B/2/4/6/10 Production |
|  | DRD2 Expression Targets |
|  | GALR1/2/3 -> POMC/NPY Production |
|  | HRH2/4 -> IL6/10 Production |
|  | CHRM1 -> IL2 Production |
|  | CHRM1/2/3 -> Vascular Motility |
|  | OPRK -> Pain Perception |
|  | PTGIR -> IL6 Production |
|  | HTR1 -> IL6 Production |
|  | HTR5 -> TNF Production |
|  | HTR1 -> Membrane Transport |
|  | CHRNA7 -> NOS1 Production |
|  | NMDA Receptors -> Ca2+/CREB Activation/PGE2 Synthesis |
|  | P2RY11/13/14 -> IL8/10 Production |
|  | Luteinizing Hormone in Oocyte Meiotic Resumption |
|  | Cardiomyocyte Hypertrophy |
|  | Hypertrophic Cardiomyopathy |
|  | Endothelin Signaling in Arterial Hypertension |
|  | Aldosterone Synthesis Acute Regulation |
|  | Smooth Muscle Cell Dysfunction in Arterial Hypertension |
|  | TRPM4/6/7/8 Signaling in Arterial Hypertension (Hypothesis) |
|  | Smooth Muscle Cell Dysfunction in Pulmonary Hypertension |
|  | Myocardial Remodeling |
|  | Contraction Due Vasospasm |
|  | Cell Cycle Dysregulation in Melanoma |
|  | Melanoma |
|  | Apoptosis Block in Melanoma Cell |
|  | MITF as a Regulator of Melanoma Cell Development |
|  | Th0-Cell Aberrant Activation in Systemic Lupus Erythematosus |
|  | Hypothyrodism Associated Hyperprolactinemia |
|  | DRD2 Pharmacological Inhibition in Hyperprolactinemia |
|  | Ethanol Induced Efects in Hyperprolactinemia |
|  | GNRH Signaling Impairment in Hypogonadotrophic Hypogonadism |
|  | Prolactinoma Overiew |
|  | EDN, NRG, NRTN, and GDNF/RET Signaling in Hirschsprung Disease |
|  | Hodgkin Lymphoma |
|  | T-Cell Acute Lymphoblastic Leukemia |
|  | Airway Smooth Muscle Cell High Rate Proliferation |
|  | IgE Induces Airway Smooth Muscle Cell Proliferation |
|  | BDNF Upregulation Triggered by Seizures |
|  | Apoptosis and Immediate Early Gene Activation in Epileptiform Disorders |
|  | Angiogenesis in Endometriosis |
|  | Coagulation Factors and PAR1/2 Receptors (F2R/F2RL1) in Endometriosis |
|  | EGFR and PTAFR Signaling in Ovarian Cancer |
|  | HPV E5 Regulates EDNRA and EGFR Signaling Pathway in Keratinocytes |
|  | VEGFA in Neovascular Glaucoma |
|  | PGF2-alpha Effect on Treatment |
|  | Plasmin Role in Glomerulonephritis |
|  | Sertoli Cells Dysfunction Causes Male Infertility |
|  | ANGPT1 -> CREB/CREBBP Expression Targets |
|  | ANGPT2 -> AP-1/CREBBP/MYC Expression Targets |
|  | AREG -> CREB/CREBBP Expression Targets |
|  | AREG -> NCOR2 Expression Targets |
|  | AREG -> SMAD1 Expression Target |
|  | AREG -> HIF1A Expression Targets |
|  | AREG -> AP-1 Expression Targets |
|  | Noradrenaline/Gs Expression Targets |
|  | Noreadrenaline/Gq Expression Targets |
|  | Epinephrine/Gs Expression Targets |
|  | Acetylcholine Expression Targets |
|  | Serotonin/Gq Expression Targets |
|  | Epinephrine/Gq Expression Targets |
|  | Dopamine/Gi Expression Targets |
|  | B-Cell Receptor -> AP-1 Signaling |
|  | BTC -> AP-1/ATF/CREB Expression Targets |
|  | BTC -> EP300/ETS/ETV/SP1 Expression Targets |
|  | SELE -> ELK-SRF Signaling |
|  | CD19 Expression Targets |
|  | CD81 -> AP-1 Expression Targets |
|  | CD247 Expression Targets |
|  | gamma Globulins Expression Targets |
|  | FCGR3A Expression Targets |
|  | CD19/CR2 Expression Targets |
|  | CD72 -> CREB/CREBBP Expression Targets |
|  | CD8 -> ATF/CREB/CREBBP Expression Targets |
|  | CD80 -> NF-kB Expression Targets |
|  | CD86 -> NF-kB Expression Targets |
|  | CXCL6 Expression Targets |
|  | CXCL1 Expression Targets |
|  | CXCL2 Expression Targets |
|  | CXCL3 Expression Targets |
|  | CXCL5 Expression Targets |
|  | CHRNA7 -> CREB Signaling |
|  | Clozapine Induced Granulocytopenia |
|  | EGF -> HIF1A Expression Targets |
|  | EGF -> AP-1/ATF Expression Targets |
|  | EGF -> CREB/CREBBP/ELK/SRF/MYC Expression Targets |
|  | EGF -> TP53 Expression Targets |
|  | EGF -> NCOR2 Expression Target |
|  | EGFR -> NCOR2 Signaling |
|  | EGFR/ERBB2 -> TP53 Signaling |
|  | ERBB2/3 -> EP300/ETS/ETV/SP1 Signaling |
|  | EGFR -> SMAD1 Signaling |
|  | EGFR/ERBB2 -> HIF1A Signaling |
|  | EGFR -> AP-1/ATF2 Signaling |
|  | EGFR -> AP-1/CREB/ELK/SRF/MYC Signaling |
|  | EREG -> CREB Expression Target |
|  | EREG -> EP300/SP1 Expression Targets |
|  | EREG -> HIF1A Expression Target |
|  | EREG -> AP-1/ATF Expression Targets |
|  | Erythropoietin -> AP-1/MYC/CREB Expression Targets |
|  | Erythropoietin -> ELK/SRF Expression Targets |
|  | PECAM1 Expression Targets |
|  | F2 -> AP-1/CREB/ELK/SRF/SP1 Expression Targets |
|  | PLG -> AP-1/CREB/ELK/SRF/SP1 Expression Targets |
|  | FGF9 -> AP-1/CREB/MYC Expression Targets |
|  | FGF10 -> AP-1/CREB/CREBBP/MYC Expression Targets |
|  | FGF1 -> AP-1/CREB/ELK/SRF/MYC Expression Targets |
|  | FGF1 -> NCOR2 Expression Target |
|  | FGF7 -> RUNX Expression Targets |
|  | FGF8 -> AP-1/CREB/MYC Expression Targets |
|  | FGF1 -> RUNX Expression Targets |
|  | FGF18 -> AP-1/CREB Expression Targets |
|  | FGF23 -> NCOR2 Expression Targets |
|  | FGF8 -> RUNX Expression Targets |
|  | FGF9 -> RUNX Expression Targets |
|  | FGF2 -> NCOR2 Expression Targets |
|  | FGF4 -> AP-1/MYC Expression Targets |
|  | FGF7 -> AP-1/CREB/CREBBP/MYC Expression Targets |
|  | FGF2 -> RUNX Expression Targets |
|  | FGF2 -> AP-1/CREB/CREBBP/ELK/SRF/MYC Expression Targets |
|  | FGFR -> RUNX2 Signaling |
|  | FGFR -> AP-1/CREB/CREBBP/ELK/SRF/MYC Signaling |
|  | ADCYAP1 Expression Targets |
|  | FSHR Expression Targets |
|  | Glutamate/Gq Expression Targets |
|  | VIP Expression Targets |
|  | HBEGF -> AP-1/ATF Expression Targets |
|  | HBEGF -> TP53 Expression Targets |
|  | HBEGF -> EP300/ETS/ETV/SP1 Expression Targets |
|  | HBEGF -> HIF1A Expression Targets |
|  | HBEGF -> CREB/MYC Expression Targets |
|  | HGF -> AP-1/CREB/ELK/SRF/MYC Expression Targets |
|  | HGFR -> AP-1/CREB/MYC Signaling |
|  | TAM Receptor Signaling Activation in Cancer |
|  | CD19 -> JUN/ELK Signaling |
|  | FcIgER -> ELK-SRF Signaling |
|  | ICAM1 -> AP-1/CREB/ELK/SRF Signaling |
|  | FibronectinR -> AP-1/ELK/SRF/SREBF Signaling |
|  | CTGF -> AP-1/CREB/MYC Expression Targets |
|  | CTGF -> NCOR2 Expression Target |
|  | ICAM1 Expression Targets |
|  | IL16 -> AP-1 Expression Targets |
|  | UrokinaseR -> ELK/SRF Signaling |
|  | PTPRC -> BCL6 Expression Targets |
|  | Prostaglandin F Expression Targets |
|  | Thromboxane A2 Expression Targets |
|  | LPA Expression Targets |
|  | PAF/Gq -> AP-1/ATF1/CREB/ERK/SRF Expression Targets |
|  | Dronabinol/Anandamide Expression Targets |
|  | Morphine Expression Targets |
|  | PAF/Gq -> NF-kB Expression Targets |
|  | S1P Expression Targets |
|  | PGE1 Expression Targets |
|  | Elevated Receptors -> Expression Targets in Lymph Node |
|  | NRG1 -> EP300/ETS/ETV/SP1 Expression Targets |
|  | NRG1 -> CREB/CREBBP/ELK/SRF/MYC Expression Targets |
|  | NRG1 -> TP53 Expression Target |
|  | NRG1 -> AP-1/ATF Expression Targets |
|  | NRG1 -> HIF1A Expression Target |
|  | NGF -> SMAD3/NF-kB Expression Targets |
|  | NGF -> AP-1/TP53/MYC Expression Targets |
|  | NGF -> FOXO/MYCN/ELK-SRF Expression Targets |
|  | BDNF Expression Targets |
|  | NTF3 Expression Targets |
|  | NTF4 Expression Targets |
|  | NGF -> CREB/CEBPB/MEF2A Expression Targets |
|  | Adenosine Expression Targets |
|  | PDGFB -> AP-1/CREB/MYC Expression Target |
|  | PDGFC -> CREB Expression Target |
|  | PDGFD -> AP-1 Expression Targets |
|  | PDGF -> AP-1/CREB/CREBBP/MYC Expression Targets |
|  | KIT -> MITF Signaling |
|  | PDGFR -> AP-1/MYC Signaling |
|  | AVP/Gs -> CREB/ELK/SRF/AP-1/EGR Expression Targets |
|  | GNRH1 Expression Targets |
|  | NTS Expression Targets |
|  | GAST Expression Targets in GAST Expression Targets |
|  | AGT -> TP53 Expression Targets |
|  | GNRH2 Expression Targets |
|  | OXT Expression Targets |
|  | POMC Expression Targets |
|  | CCL11 Expression Targets |
|  | EDN1 Expression Targets |
|  | AGT -> CREB Expression Targets |
|  | NPY Expression Targets |
|  | IFNA1/Gq Expression Targets |
|  | AGT -> ELK/SRF Expression Targets |
|  | TAC1 Expression Targets |
|  | EDN3 Expression Targets |
|  | AVP/Gq -> CREB/ELK/SRF/AP-1/EGR Expression Targets |
|  | PDCD1 -> AP-1 Expression Targets |
|  | PTPRC -> BCL6 Signaling |
|  | AngiopoietinR -> AP-1 Signaling |
|  | GDNF -> HSF1 Signaling |
|  | NTRK -> AP-1/CREB/ELK/SRF/MYC/SMAD3/TP53 Signaling |
|  | GAS6 -> AP-1/CREB Expression Targets |
|  | FIGF -> AP-1 Expression Target |
|  | FIGF -> NCOR2 Expression Target |
|  | KITLG -> AP-1/CREB/CREBBP/MYC Expression Targets |
|  | GDNF -> HSF1 Expression Targets |
|  | CSF1 -> AP-1/CREB/CREBBP/MYC Expression Targets |
|  | PGF -> AP-1/CREB/CREBBP/MYC Expression Targets |
|  | FLT3LG -> AP-1/CREB/CREBBP Expression Targets |
|  | Lysophosphatidic Acid/LPARs Signaling |
|  | Ras-GRF Regulation Signaling |
|  | AMPK Signaling |
|  | AXL Receptor Tyrosine Kinase Signaling |
|  | KRAS Signaling |
|  | HRAS Signaling |
|  | TCR -> AP-1 Expression Targets |
|  | TGFA -> TP53 Expression Targets |
|  | TGFA -> HIF1A Expression Targets |
|  | TGFA/AP-1/ATF Expression Targets |
|  | TGFA -> CREB/CREBBP/ELK-SRF/MYC Expression Targets |
|  | Thrombopoietin -> AP-1/CREB/CREBBP/MYC Expression Targets |
|  | ROS in Triggering Vascular Inflammation |
|  | ROS and RNS in Vasoconstriction and Vasodilation Regulation |
|  | ROS in Angiotensin Mediated Cardiovascular Remodeling and Hypertrophy |
|  | PLAU -> ELK-SRF/AP-1 Expression Targets |
|  | VEGFA -> ATF/CREB/ELK-SRF Expression Targets |
|  | VEGFA -> NFATC Expression Targets |
|  | VEGFC -> ATF Expression Target |
|  | VEGFA -> AP-1/CREBBP/MYC Expression Targets |
|  | VEGFA -> NCOR2 Expression Target |
|  | VEGFR -> NFATC Signaling |
|  | VEGFR -> AP-1/CREB/MYC Signaling |
|  | VEGFR -> ATF/CREB/ELK-SRF Signaling |
|  | Model of PPARG signaling in psoriasis |
|  | Model of PPARG related pathways in psoriasis (short version) |
|  | 2\_Cardiomyocyte hypertrophy |
|  | 2\_The intense proliferation and contractile activity of smooth muscle cells in pulmonary hypertension |
|  | 2\_1\_Eosinophilia and neutrophilia in asthma: eosinophil migration and activation |
|  | 4\_1\_Airway smooth muscle cells contraction |
|  | public list |
|  | KRAS->RAFs->MAPK1/3 |
|  | NRAS->RAFs->MAPK1/3 |

---

|  |  |
| --- | --- |
| Group | Genes with Mutations Associated with Osteopetrosis |

---

|  |  |
| --- | --- |
| MedScan ID | 10125 |

---

|  |  |
| --- | --- |
| LocusLink ID | 10125 |
|  | 19419 |
|  | 29434 |

---

|  |  |
| --- | --- |
| Alias | Rasgrp I |
|  | CALDAG-GEFI |
|  | CALDAG-GEFII |
|  | MGC129998 |
|  | RAS guanyl nucleotide-releasing protein 1 |
|  | RAS guanyl releasing protein 1 |
|  | MGC129999 |
|  | Rasgrp1 |
|  | RP23-73I19.3 |
|  | RASGRP |
|  | OTTMUSP00000016207 |
|  | RAS guanyl releasing protein I |
|  | hRasGRP1 |
|  | CalDAG-GEF1 |
|  | calcium- and diacylglycerol-regulated guanine nucleotide exchange factor II |
|  | Calcium and DAG-regulated guanine nucleotide exchange factor II |
|  | guanine nucleotide exchange factor, calcium- and DAG-regulated, Rap1A |
|  | RAS guanyl releasing protein 1 (calcium and DAG-regulated) |
|  | RAS guanyl nucleotide-releasing protein I |
|  | ras activator RasGRP |
|  | RAS guanyl-releasing protein 1 |
|  | V |

---

|  |  |
| --- | --- |
| GO ID | 0005088 |
|  | 0005509 |
|  | 0019992 |
|  | 0005085 |
|  | 0008289 |
|  | 0031210 |
|  | 0001786 |
|  | 0042803 |
|  | 0008270 |
|  | 0000165 |
|  | 0007265 |
|  | 0090630 |
|  | 0030154 |
|  | 0001816 |
|  | 0002437 |
|  | 0043303 |
|  | 0070374 |
|  | 0043547 |
|  | 0046330 |
|  | 0043406 |
|  | 0046579 |
|  | 0033089 |
|  | 0032725 |
|  | 1902715 |
|  | 0032825 |
|  | 0045954 |
|  | 0001934 |
|  | 0032760 |
|  | 0051259 |
|  | 0070372 |
|  | 1900744 |
|  | 0014066 |
|  | 1900274 |
|  | 0032252 |
|  | 0007165 |
|  | 0047496 |
|  | 0005794 |
|  | 0000139 |
|  | 0005829 |
|  | 0005789 |
|  | 0042629 |
|  | 0016020 |
|  | 0005886 |
|  | 0046872 |
|  | 0035556 |
|  | 0032816 |
|  | 0007264 |
|  | 0005737 |
|  | 0005783 |
|  | 0005096 |
|  | 0017034 |
|  | 0043087 |
|  | 0038095 |
|  | 0032862 |
|  | 0007596 |
|  | 0045087 |
|  | 0030168 |
|  | 0032856 |
|  | 0051056 |
|  | 0005623 |
|  | 0005622 |
|  | 0032854 |

---

|  |  |
| --- | --- |
| KEGG ID | rno:29434 |
|  | hsa:10125 |
|  | mmu:19419 |

---

|  |  |
| --- | --- |
| Organism | Homo sapiens {Organism urn:agi-taxid:9606} |
|  | Mus musculus {Organism urn:agi-taxid:10090} |
|  | Rattus norvegicus {Organism urn:agi-taxid:10116} |
|  | Homo sapiens |
|  | Mus musculus |
|  | Rattus norvegicus |

---

|  |  |
| --- | --- |
| Mouse chromosome position | 2 59.19 cM |

---

|  |  |
| --- | --- |
| OMIM ID | 603962 |

---

|  |  |
| --- | --- |
| Rat chromosome position | 3q35 |
|  | 3q34-q35 |

---

|  |  |
| --- | --- |
| Hugo ID | 9878 |
|  | HGNC:9878 |

---

|  |  |
| --- | --- |
| Human chromosome position | 15q14 |

---

|  |  |
| --- | --- |
| Swiss-Prot Accession | B2RA89 |
|  | O95267 |
|  | O95267.2 |
|  | Q9Z1S3 |
|  | Q9Z1S3.1 |
|  | Q9R1K8 |
|  | Q9R1K8.1 |
|  | O88469 |
|  | Q56CZ0 |
|  | Q58G75 |
|  | Q59HB1 |
|  | Q5I3A8 |
|  | Q6GV31 |
|  | Q6NX39 |
|  | Q7LDG6 |
|  | Q9UI94 |
|  | Q9UNN9 |
|  | Q3URH0 |
|  | Q3V401 |
|  | Q8BQP6 |
|  | F5H514 |

---

|  |  |
| --- | --- |
| GenBank ID | NC\_000015 |
|  | XM\_017021860 |
|  | XP\_016877349 |
|  | XM\_005254114 |
|  | XP\_005254171 |
|  | XM\_011521151 |
|  | XP\_011519453 |
|  | NM\_001306086 |
|  | NP\_001293015 |
|  | NM\_001128602 |
|  | NP\_001122074 |
|  | NM\_005739 |
|  | NP\_005730 |
|  | NG\_023268 |
|  | AC116158 |
|  | AC124306 |
|  | CH471125 |
|  | EAW92372 |
|  | KF456047 |
|  | AA742351 |
|  | AB208848 |
|  | BAD92085 |
|  | AF081195 |
|  | AAC79699 |
|  | AF081197 |
|  | AAF21898 |
|  | AF106071 |
|  | AAC97349 |
|  | AK295097 |
|  | BAG58133 |
|  | AK314091 |
|  | BAG36786 |
|  | AK316067 |
|  | BAH14438 |
|  | AY634315 |
|  | AAT47482 |
|  | AY858556 |
|  | AAW32406 |
|  | AY954625 |
|  | AAX54699 |
|  | AY966005 |
|  | AAX76907 |
|  | BC032849 |
|  | BC067298 |
|  | AAH67298 |
|  | BC109296 |
|  | AAI09297 |
|  | BC109297 |
|  | AAI09298 |
|  | BU431038 |
|  | DA993002 |
|  | O95267 |
|  | NC\_000068 |
|  | NM\_011246 |
|  | NP\_035376 |
|  | AL844579 |
|  | AL928959 |
|  | CH466519 |
|  | EDL27897 |
|  | AF106070 |
|  | AAC97348 |
|  | AK013548 |
|  | AK028308 |
|  | BAE20439 |
|  | AK047613 |
|  | BAC33100 |
|  | AK134005 |
|  | AK141524 |
|  | BAE24718 |
|  | BC057120 |
|  | AAH57120 |
|  | BC057341 |
|  | AAH57341 |
|  | BE991595 |
|  | Q9Z1S3 |
|  | NC\_005102 |
|  | NM\_019211 |
|  | NP\_062084 |
|  | AC\_000071 |
|  | AABR07053473 |
|  | AAHX01024503 |
|  | AAHX01024504 |
|  | CH473949 |
|  | EDL79846 |
|  | AF060819 |
|  | AAC40137 |
|  | AF081196 |
|  | AAC79700 |
|  | Q9R1K8 |
|  | XM\_011521152 |
|  | XP\_011519454 |
|  | XR\_931740 |
|  | XM\_011521154 |
|  | XP\_011519456 |
|  | XM\_011521153 |
|  | XP\_011519455 |
|  | NC\_018926 |
|  | AMYH02030216 |
|  | EU832766 |
|  | ACE87777 |
|  | GQ129309 |
|  | ACT64443 |
|  | AC\_000024 |
|  | AAHY01019867 |
|  | AAHY01019868 |
|  | AAHY01019869 |
|  | AAHY01019870 |
|  | AAHY01019871 |
|  | AAHY01019872 |
|  | AC\_000147 |
|  | ABBA01038478 |
|  | ABBA01038479 |
|  | XM\_005254113 |
|  | XP\_005254170 |
|  | XM\_005254116 |
|  | XP\_005254173 |
|  | XM\_005254115 |
|  | XP\_005254172 |
|  | AABR06025722 |
|  | AABR06025723 |
|  | AABR06025724 |

---

|  |  |
| --- | --- |
| Swiss-Prot ID | GRP1\_RAT |
|  | GRP1\_HUMAN |
|  | GRP1\_MOUSE |

---

|  |  |
| --- | --- |
| Cell Localization | Cytoplasm |
|  | Cytosol |
|  | Cell membrane |
|  | Golgi apparatus membrane |
|  | Endoplasmic reticulum membrane |
|  | Cytoplasm, cytosol |

---

|  |  |
| --- | --- |
| Ensembl ID | ENSG00000172575 |
|  | ENSP00000388540.2 |
|  | ENST00000450598.6 |
|  | ENSP00000452721.1 |
|  | ENST00000559830.5 |
|  | ENSP00000310244.5 |
|  | ENST00000310803.10 |
|  | ENSP00000453583.1 |
|  | ENST00000558432.5 |
|  | ENSMUSG00000027347 |
|  | ENSMUSP00000099593.4 |
|  | ENSMUST00000102534.10 |
|  | ENSRNOG00000005404 |
|  | ENSRNOP00000007687.4 |
|  | ENSRNOT00000007687.4 |
|  | ENSP00000388540 |
|  | ENST00000450598 |
|  | ENSP00000310244 |
|  | ENST00000310803 |
|  | ENSP00000452721 |
|  | ENST00000559830 |
|  | ENSP00000413105 |
|  | ENST00000414708 |
|  | ENSMUSP00000099593 |
|  | ENSMUST00000102534 |
|  | ENSRNOP00000007687 |
|  | ENSRNOT00000007687 |

---

|  |  |
| --- | --- |
| MGI ID | MGI:1314635 |
|  | 1314635 |

---

|  |  |
| --- | --- |
| RGD ID | 3539 |

---

|  |  |
| --- | --- |
| Unigene ID | Hs.591127 |
|  | Mm.42150 |
|  | Rn.162685 |

---

|  |  |
| --- | --- |
| Homologene ID | 4195 |

---

|  |  |
| --- | --- |
| Shape | Stick-vertex |

---
